# Gut Microbiota Parameters Potentially Useful in Clinical Perspective

**DOI:** 10.3390/microorganisms9112402

**Published:** 2021-11-22

**Authors:** Francesco Di Pierro

**Affiliations:** 1 Digestive Endoscopy & Gastroenterology, Fondazione Poliambulanza, 25124 Brescia, Italy; f.dipierro@vellejaresearch.com; 2 UNICAM, Camerino University, 62032 Camerino, Italy; 3 Scientific Department, Velleja Research, 20124 Milan, Italy

**Keywords:** human gut microbiota, Firmicutes/Bacteroidetes ratio, *Prevotella*/*Bacteroides* ratio, Gram-positive/Gram-negative ratio, *Fusobacterium*, *Faecalibacterium*, biomarkers

## Abstract

Interest in gut microbiota analyses is at an all-time high. Gut microbiota is thought to relate to an increasing range of diseases of interest to physicians and nutritionists. Overweight, obesity, response to diet, metabolic syndrome, low grade inflammation, diabetes and colon neoplasms could maybe be observed in microbiota if affordable markers were available. Possible biomarkers like the Firmicutes/Bacteroidetes ratio, the Gram-positive/Gram-negative ratio, the *Prevotella*/*Bacteroides* ratio, and the *Fusobacterium nucleatum*/*Faecalibacterium prausnitzii* ratio are here reviewed in a narrative way in the attempt to highlight their possible future role in routine practice and clinically relevant diagnostics.

## 1. The Search for Biomarkers

The logic underlying the analysis of microbiota coincides with the possibility of intercepting parameters being interpreted as indicators, at least putative, of some deformity compared to a standard. As demonstrated by many authors, this precise need could be met by the biodiversity [1,2,3,4,5,6,7,8,9], the size and composition by genus [10,11,12,13,14,15,16] and the classification by enterotypes [17,18,19]. However, scientific research has identified the existence of other possible markers useful in the interpretation of the fecal bacterial consortium. Among these, the potentially most useful ones could be: the Firmicutes/Bacteroidetes ratio (ratio between phyla), the Gram-positive/Gram-negative ratio (ratio between groups of taxa), the *Prevotella*/*Bacteroides* ratio (ratio between genera) and the *Fusobacterium nucleatum*/*Faecalibacterium prausnitzii* ratio (ratio between species).

The existence of these markers, however, perhaps because they are sometimes considered only putative, has not translated into any application of practical health usefulness and is not currently taken into consideration by the health authorities. Probably the partial lack of longitudinal studies capable of following these parameters over time, the current lack of a precise standardization of genomic sequencing methods, the waiting times necessary for laboratories to obtain well-filtered results with a high sequence homology and costs analytics which for now are still relatively high, or in any case not within everyone’s reach, contribute to creating this possible gap. Despite this, however, a careful examination of the available scientific literature shows that sometimes the lack of clinical use of these parameters is also perhaps the result of an incorrect interpretation of their value. Take for example the case of the Firmicutes/Bacteroides ratio. As it will be shown in this review, at least from a narrative point of view commonly perceived also at a scientific and congressional level, the parameter, when high, would seem to indicate the obese phenotype and, when reduced, a lean phenotype. If this interpretation also corresponded to reality, interpreted in this way the parameter would be of no use. In fact, to identify obese or lean subjects, the eyes and at most a scale are enough. Analysis of the fecal microbiota allows us to have a picture only of the colonic and luminal bacterial consortium. It therefore shows the identity of those bacteria involved in the terminal processes of digestion of food residues present in the colon. The process of absorption of food and calories, on the other hand, takes place mainly in the small intestine. Thus, from a caloric point of view, the information that can be obtained is rather modest. Despite this, it is not useless. In fact, the colonic microbiota can suggest whether, and with what degree of completeness, the undigested fiber present in the colon will be metabolized with the production of short-chain fatty acids (SCFA). The latter cannot be considered zero calories, and their direct analysis, performed by sampling the stool, risks giving inaccurate results as they are constantly produced and immediately absorbed compounds. In this sense, however, the Firmicutes/Bacteroidetes ratio provides, as will it be shown, a measure of the exhaustive metabolization capacity of the calories ingested by the subject and present only in the colon. As it will be shown, these correspond to approximately 100–200 kcal/day. An irrelevant value for the obese patient only if not considered cumulatively over time (months and years). Likewise, the same is true for the Gram-positive/Gram-negative ratio. This is considered a parameter proportional to the presence of bacterial lipopolysaccharide (LPS). As it will be shown, this is partly wrong. Some Gram-negatives, indicated as such for example in the metagenomic databases, do not have LPS (for example, Tenericutes). Others, like *Bacteroides*, have an LPS in their membrane thousands of times less pro-inflammatory than the standard one (*E. coli*). In this sense, therefore, the inflammatory potential mediated by the LPS detected via the sequencing of the genomes of colonic bacteria will have to be interpreted considering these aspects as well. In the same way, other parameters like the ratio between *Prevotella* and *Bacteroides* or the simple detecting of *Fusobacterium*, which can be easily investigated, present specific criticalities that need to be discussed to obtain, as far as possible with the information available today, the most correct interpretation possible.

## 2. The Firmicutes/Bacteroidetes Ratio

The colonic fecal microbiota is basically composed of seven main phyla: Firmicutes, Bacteroidetes, Proteobacteria, Actinobacteria, Verrucomicrobia, Fusobacteria and Tenericutes. Firmicutes and Bacteroidetes, in a very consistent way, come to represent, in healthy subjects, approximately 90–95% of the entire consortium [20]. In no other ecosystem do these two *phyla* manifest themselves in such a dominant way. This shows that the colon is really their home and that here the two *phyla* perhaps coexist without too much competition and, on the contrary, probably manifesting aspects of cooperation or specialization. Although their relative summation corresponds to approximately 90–95% of the entire microbiota, within this value the contribution of one and the other is instead variable, and the increase of one corresponds to the decrease of the other. In this sense, the ratio between the two, that is the percentage value of the first (Firmicutes) divided by the percentage value of the second (Bacteroidetes), can virtually go from infinity to zero. This ratio generally fluctuates from 0.1 to 10 (when it is 0.1 the Firmicutes are 1/10 of the Bacteroidetes, when it is 10 the Firmicutes are ten times the Bacteroidetes) even if, more frequently, a narrower range of ratios is highlighted, approximately between 0.2 and 4, with values between 0.8 and 1.2, which can be considered as a possible reference [20]. It therefore becomes interesting to try to attribute a translational, clinical meaning to the observable relationships. Indeed, in 2005, a group from Washington University first observed something similar [21]. Comparing, in genetically obese mice (*ob*/*ob*) and in mice with the lean phenotype (*ob*/+ and +/+), the relative percentage of these two *phyla*, a statistically significant correlation was observed between the expression of a higher percentage of Firmicutes and the obese phenotype and, conversely, between a higher percentage of Bacteroidetes and the lean phenotype. In particular, the obese mice had a profile in Firmicutes that was on average 20% higher, with an identical reduction in Bacteroidetes. The Firmicutes/Bacteroidetes ratio (hereinafter referred to simply as F/B) was slightly lower than 4 in obese mice and approximately 1.5 in those with a lean phenotype. Note that, exactly as in humans, the mouse has a percentage value referable to the sum of Firmicutes + Bacteroidetes of approximately 90–95%. Since the analysis of the fecal microbiota “visualize” almost exclusively the colonic bacteria and in consideration of the fact that the colon absorbs, in nutritional terms, only short-chain fatty acids, mainly acetate, propionate and butyrate, the same research group analyzed the content of the latter in the excreted fecal material [22]. While taking into consideration the possible inaccuracy of this detection (measuring the residual fecal content of short-chain fatty acids does not absolutely indicate how much was produced but only how much is left; however, it can be assumed that the more there is recovered the more there was produced), in genetically obese mice they observed a significant greater presence of acetate and butyrate, but not propionate. Since Bacteroidetes mainly produce propionate and Firmicutes, especially starting from acetate, mainly butyrate, the values of acetate and butyrate are therefore often in parallel, and this data evidently correlated well with the shift, in favor of the Firmicutes, observed. It is noteworthy that they found approximately 10% more calories in the fecal material of lean mice than observed in the stool of the obese ones. In obese mice, therefore, the capacity of caloric extraction, obtainable from the catabolism of the residual colonic fibers, appeared to be more efficient than it was in lean mice. Finally, the transplantation of fecal material, with sampling performed from mice with an obese phenotype (*ob*/*ob*), determined, in the recipient animal, the detection of a percentage of body fat higher than that which was determined with the transplantation of stool from mice with a lean phenotype (+/+). The results were certainly consistent, but clinical evidence was still lacking. This came in December 2006 when the same group observed the progressive variation of the F/B ratio, with decrease of Firmicutes and corresponding growth of Bacteroidetes, in 12 obese patients subjected to 52 weeks of respectively low-calorie and low-sugar or low-calorie and low-fat diets [23]. Throughout the period, each subject’s microbiota remained more like itself than changing to become more similar to the other subjects’ microbiota. Richness remained almost constant in all subjects and the correlation between weight loss and decreased F/B ratio became evident in subjects on a low-sugar diet when the weight lost exceeded 2% of the initial weight and, in subjects on a low-fat diet, when the weight lost had exceeded 6% of that recorded at enrollment. The relative reduction observed in Firmicutes, parallel to the progressive weight loss, concerned the entire *phylum* and was not attributable to the reduction and/or disappearance of any bacterial group. The same was observed with respect to the percentage growth of Bacteroidetes.

**Figure 1:**
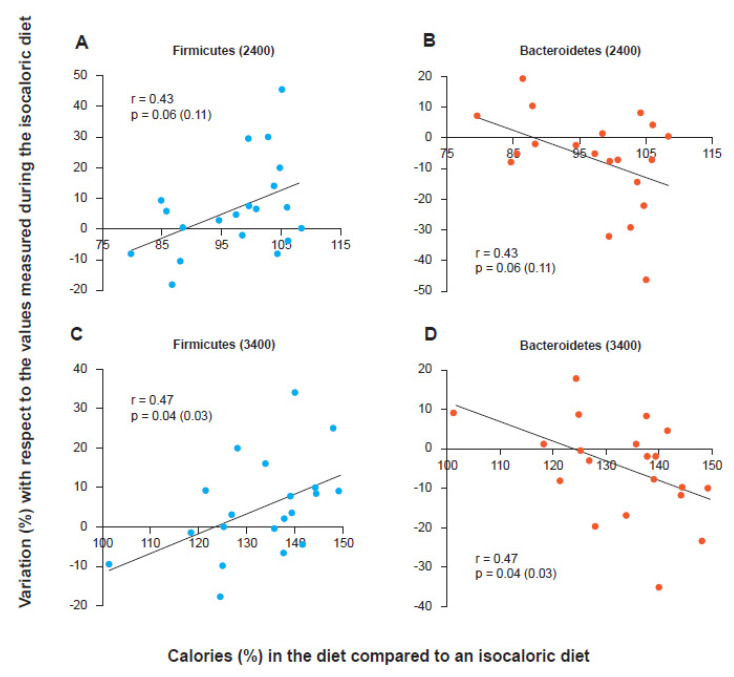
Firmicutes and Bacteroidetes trends as the calories in the diet vary with respect to an isocaloric condition for the individual regardless of their phenotype (obese or lean). Firmicutes variation with 2400 kcal/day (**A**) and 3400 kcal/day (**C**) diets. Bacteroidetes variation with diet at 2400 kcal/day (**B**) and at 3400 kcal/day (**D**) [30].

Although most subsequent studies have actually shown, both in children and in adults and the elderly, the existence of a significant correlation between a high F/B ratio and the occurrence of an obese phenotype [24,25,26], as well as a significant correlation between progressive decrease in the F/B ratio and progressive weight loss obtained both with the low-calorie diet [27] and with bariatric surgery [28], some authors have reported that both the methodological differences in the management of stool and in sequencing techniques and the poor characterization of obese subjects enrolled in the studies and the total non-consideration of the many other variables capable of influencing the taxonomic aspects of a microbial consortium (antibiotics, fiber content in the diet, colonic transit, etc.) could contribute to making this correlation sometimes unobservable and therefore prevent us from considering the F/B ratio as a really reliable marker [29]. It could be that the issue is misplaced. In fact, it is not necessary to have an obesity marker, even if having it as “bacterial” would be very original. To identify an obese person it is only necessary to look at them. What could be interesting is having a marker that indicates the predisposition to gain or lose weight and that allows the physician or the nutritionist to follow the progress of this predisposition during a low-calorie diet. Indeed, early publications did not discuss the F/B ratio as a “method of intercepting obese people”. Instead, they described the trend of the F/B ratio during a low-calorie diet capable of producing weight loss. These publications concluded by arguing that the phenomenon was mainly linked to the presence of a “more or less wasteful” microbiota, that is, a bacterial consortium capable, or not, of losing calories with stool. In fact, if this were really the mechanism, the variation of the F/B ratio would not be observed only in the progressive weight loss of the obese. The phenomenon, in an opposite way, should for example be observable even in the lean when chronically subjected to a high-calorie diet. Indeed, by simultaneously enrolling subjects with a lean phenotype and subjects with an obese phenotype and subjecting them to both low-calorie, isocaloric and high-calorie diets, it was possible to observe how the phenomenon of the variation of the F/B ratio is independent from the starting phenotype but instead strictly dependent only on the caloric balance administered, that is, from the nutritional load calculated in relation to the real caloric needs of the individual [30]. In fact, every subject, underweight, normal weight or obese, has an isocaloric balance that gives them their “constant weight”. Each subject also has a low-calorie balance that determines their weight loss and a high-calorie balance that instead produces an increase. It is this aspect, the nutritional load compared to the needs, that determines the variation of the F/B ratio, upwards or downwards. Although this view may not be accepted by everyone or could be considered by some an oversimplification, weight loss obtained via dieting corresponds to a chronic negative energy state in which individuals consume much less than the balance that would allow them to maintain a constant weight, regardless of their phenotype. The most meaningful hypothesis therefore is that the association between the observed taxonomic changes and weight loss reflects the effects of a reduction in the nutritional load rather than weight loss. The results confirmed this hypothesis. Both lean and obese subjects were enrolled (and hospitalized to be able to precisely control the calories in input, diet, and those in output, stool and urine). The isocaloric value for the inpatients was calculated. Then, both obese and lean subjects were fed with two diets, identical from a qualitative point of view (24% protein, 16% fat and 60% carbohydrate and with the same fiber value) but different in terms of calories: 2400 and 3400 kcal/day. As shown in Figure 1, the trend of Firmicutes and Bacteroidetes was clearly demonstrated to be related to the nutritional load with respect to the isocaloric value. The subjects, both lean and obese, who ate more than their isocaloric value (in the graph the cut off corresponds to the value 100 on the abscissa) increased the relative content in Firmicutes and at the same time decreased that of Bacteroidetes. The weight gain proved to be linked to the lack of “waste” of calories, and in fact, an increase of about 20% in Firmicutes, with a corresponding decrease in Bacteroidetes, corresponded to an increase in the stool caloric collection capacity, that is, calories that were not excreted with the feces, by about 150 kcal. This value turned out to be independent of the calories ingested in the obese (who therefore appear better “built” not to lose calories) but proved to be dependent on calories when the diet was administered to the lean ones. These results could suggest the possibility that the microbiota “perceives” the availability of nutrients and favors their absorption or not. The role of a calorie-sensing colonic microbiota is certainly fascinating. More likely, however, the explanation is of a chemical-mechanical nature and could be linked to the fact that the more “waste” is not absorbed in the small intestine the more there is availability of material to ferment in the colon. However, fermentation produces short-chain fatty acids, and these contribute to slightly lowering the colonic pH. The Bacteroidetes, unlike the Firmicutes, seem to not tolerate well a pH not close to neutrality. Both Firmicutes and Bacteroidetes can ferment the colonic material, but while the former continues to do so even when the pH drops below neutrality (even slightly), the latter “slow down” because they cannot stand a pH below 6.5 [31]. In conclusion, the F/B ratio cannot be considered as a marker of obesity, but it could be used to evaluate a predisposition to lose, or not, colonic calories, and in a longitudinal way, it could be used to follow the trend of the gut microbiota during a low-calorie diet, knowing that the more this ratio drops the more the colonic microbiota will lose in performance as regards to the exhaustive absorption of calories. Evaluating the F/B ratio before assuming a low-calorie diet could then allow better decisions regarding a precise value in kcal/day to set. Specifically, after evaluating the basal calorimetry, the extra daily energy expenditure, and the weight target to be reached, an F/B value increased by 30% compared to the control would perhaps suggest a further reduction of 225 kcal/day, to be calculated as absorbable calories in the small intestine (starches for example). This is because, as shown, for every 20% more Firmicutes, the “capture” of about 150 colonic kcal should be assumed, otherwise, they are excreted with the stool. Moreover, longitudinally, the continuous observation of the F/B ratio could instead suggest a change in progress in the diet. In fact, the F/B value tends to describe only a lower or greater propensity not to miss any colonic calories. “Reading” this propensity would therefore allow a more precise nutritional attitude also during the patient’s management.

## 3. Active Role of Firmicutes in the Predisposition to Weight Gain

**Figure 2:**
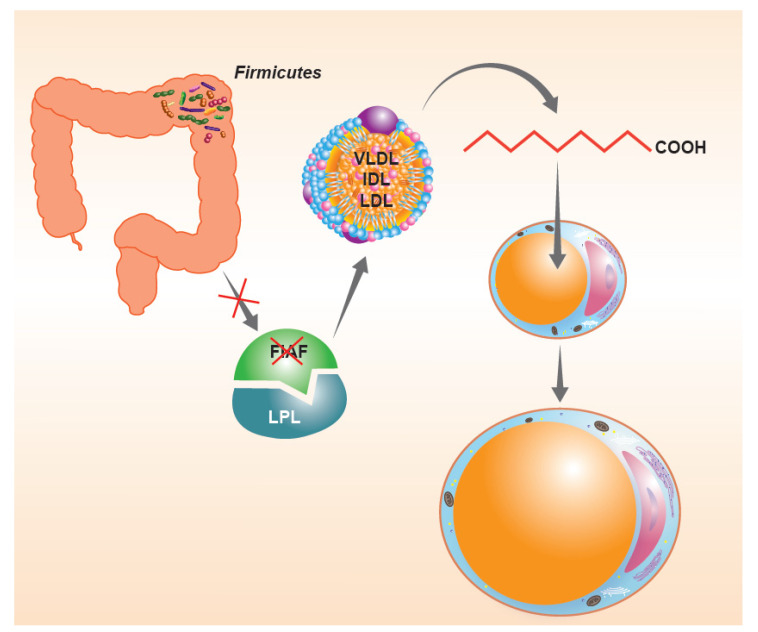
The suppression of intestinal expression of a lipoprotein with inhibitory functions on intestinal lipase (LPL), the so-called fasting-induced adipose factor (FIAF) induced by Firmicutes, would increase the activity of LPL producing an increase in fatty acid extraction capacity from post-prandial fat particles, with an increase in the performance of fat accumulation within the adipocytes.

In consideration of what has been described so far, the intestinal microbiota, when particularly rich or unbalanced in favor of the *phylum* Firmicutes, seems to be an important “microbial” factor capable of influencing the energy balance of the host. For a long time, there have been questions about the possible mechanisms responsible for this phenomenon. As anticipated before, the tolerance of a lower pH would facilitate the Firmicutes in the continuous fermentation of the residual fiber to short-chain fatty acids. However, there are also other possible “whys”, perhaps a little more biochemical. The first would seem to involve the suppression of intestinal expression of a lipoprotein with inhibitory functions on intestinal lipase (LPL), known as the fasting-induced adipose factor (FIAF). The suppression of the FIAF induced by intestinal bacteria (Figure 2), mainly when belonging to the *phylum* Firmicutes, would in fact increase the activity of LPL, producing an increase in the capacity of extraction of fatty acids from post-prandial fat particles, primarily chylomicrons, with an increase in the performance of fat accumulation within the adipocytes [32]. A second mechanism, capable of determining changes in the energy balance of the host, would appear to be connected precisely with the ability to extract residual energy from the fecal material described above. Much of the high molecular weight fiber ingested with the meal in fact escapes the host’s glycosidases, but can be effectively metabolized by colonic bacterial populations, with relative production of short-chain fatty acids, mainly acetate, propionate, and butyrate [33]. The colonic epithelium derives 60–70% of the energy necessary to survive from the simple presence of short-chain fatty acids produced by the microbiota, mainly butyrate [34,35]. The presence of important relative Firmicutes values can lead to an increase in catabolic capacity with an increase in the production of local butyrate values such that they are able to “feed” the entire enterocyte population. The phenomenon obviously could lead to a lack of use of 30–40% of calories that the enterocyte should instead derive from the systemic circulation. These calories are obviously not “wasted” but stored in the form of body fat. Conversely, when Bacteroidetes are relatively dominant, the most produced fiber is propionate. This short-chain fatty acid is not absorbed by the enterocyte but passes through it and ends up entering the cycle of hepatic gluconeogenesis, probably fueling the phenomenon of satiety and, in the long run, insulin resistance [36,37]. Then, there is a third aspect that deserves consideration. In fact, it is possible to investigate the metabolic preferences, a reflection of the gene contribution alone, of the various *phyla* [38]. From this point of view, they are in fact quite different from each other. For example, *Enterobacteriaceae* are preferentially proteolytic and lipolytic. Actinobacteria are mostly saccharolytic. Firmicutes, on the other hand, tend to be “generalists”, that is, efficient in proteolytic, saccharolytic and lipolytic demolition practically in equal measure. It is therefore easy to understand how this can be disadvantageous in a modern, western and rich society, where food abundance is the rule. Whatever the host gives them to eat, the Firmicutes “eat”.

## 4. Metabolic Endotoxemia

**Figure 3:**
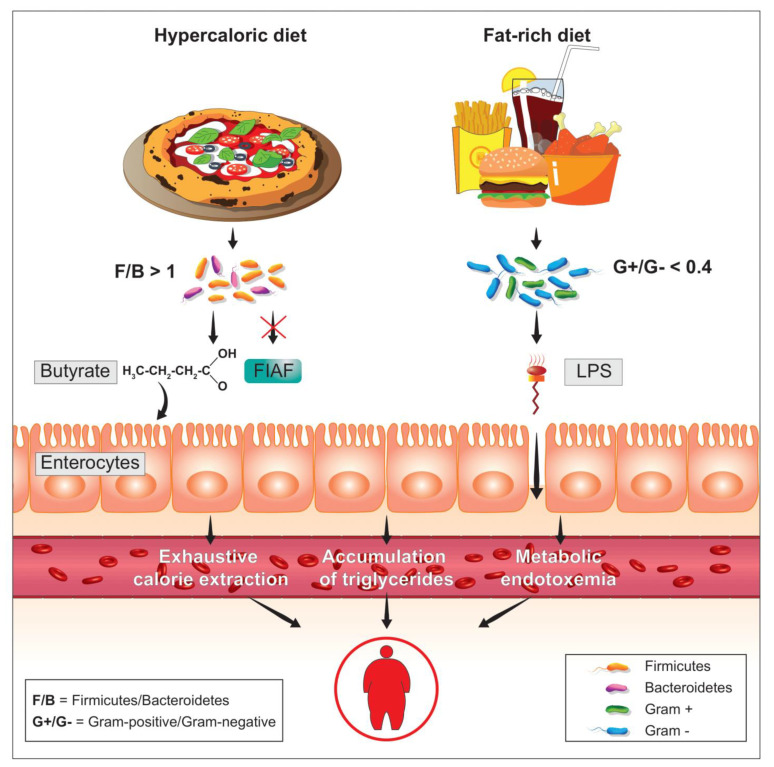
In relation to a high level of LPS, able to overcome the enterocyte barrier thus reaching the plasma circulation, phenomena such as metabolic endotoxemia can be generated, which are called into question to explain some forms of obesity coupled with metabolic syndrome. An increased percentage of Gram-negative bacterial species together with a condition of high intestinal permeability could favor this phenomenon.

As it is well-known, diabetes and obesity are two metabolic diseases characterized (also) by insulin resistance and low-grade inflammation. Looking for an inflammatory factor responsible for the onset of insulin resistance, obesity, and diabetes, a few years ago [39] the bacterial lipopolysaccharide, more commonly known as LPS, was identified as a possible trigger (Figure 3). It was found that the normal presence of LPS in plasma increased or decreased respectively during the feeding and fasting phases and that a high-fat diet, pursued for at least four weeks, increased the plasma concentration of LPS by about three times, producing an inflammatory state which was identified as metabolic endotoxemia. This inflammatory state was caused by the increase in the intestinal populations of Gram-negative bacteria, in whose membrane LPS is present, caused by the diet rich in dietary fat. In mice, the subcutaneous infusion, lasting four weeks, of quantities of LPS proportional to those observed in metabolic endotoxemia of food origin, caused increases in fasting blood glucose and insulin with evident weight gain. Liver and adipose tissue increased in weight similarly to what was observed in mice fed a diet high in dietary fat. In the liver of these animals, the induction of strong insulin resistance, especially hepatic, was also highlighted. The main peripheral receptor for LPS is CD14 [40]. From the LPS-CD14 interaction, cells such as the macrophage release TNF-α (tumor necrosis factor-α), a powerful mediator of inflammation [41]. The metabolic endotoxemia, triggered by this interaction, and by the subsequent release of TNF-α, was inferred from the fact that genetically CD14-deficient mice were instead resistant to most of the metabolic diseases induced by both subcutaneous LPS infusion and a high-fat diet. These data clearly demonstrate how metabolic endotoxemia produces an increase in systemic inflammatory tone and triggers reactions capable of producing obesity and diabetes, underlying the phenomenon of insulin resistance. In this sense, therefore, reducing the plasma concentration of LPS by negatively influencing the percentage presence of Gram-negative colonial species could prove to be a powerful strategy for the control of metabolic diseases.

## 5. Gram-Positive and Gram-Negative

From a structural point of view, Gram-positive bacteria are bacteria that have a lipid bilayer very similar to that of eukaryotic cells, with proteins also above all trans-membrane, with fractions of cholesterol aimed at reducing the excessive membrane fluidity but also with an important external thickening consisting of a mixed protein-sugar matrix, known as peptidoglycan (or bacterial mucopeptide or murein), which further strengthens and shields the thin membrane of the bacterium from the external environment [42]. Structurally, peptidoglycan is a polymer consisting of long linear polysaccharide chains, joined together by cross-links determined by the presence of amino acid residues. Polysaccharide chains are composed of the repetition of a disaccharide, which in turn consists of two monosaccharides, *N*-acetyl-glucosamine (or NAG) and *N*-acetyl-muramic acid (or NAM), linked together by glycosidic bonds of type β-1,6. The disaccharides are then linked to each other with β-1,4 type glycosidic bonds. Linked to each molecule of NAM there is a penta-peptide that ends with two equal amino acids in the dextrorotatory form, more frequently with two molecules of D-alanine. It is precisely these terminal D-alanine which, thanks to the action of the transpeptidase enzyme, allow the formation of cross-links within the peptidoglycan. More precisely, the transpeptidase originates a peptide bond between the third amino acid of a polysaccharide chain and the fourth amino acid of the parallel chain [43]. The result is a uniform and relatively thick cell wall (20–80 nm) made up of numerous layers of peptidoglycan which are intersected by teichoic acids (polymers of alcohols and phosphates) [44]. The Gram-positive wall is very polar and allows the permeation of hydrophilic molecules, such as those that make up the Gram stain (Gram staining is a chemical process, based on crystal violet and acetone/alcohol, for coloring the microorganisms, which was invented in 1884 by a Danish bacteriologist, Hans Christian Gram, from whom the technique takes its name). The Gram-negatives, on the other hand, above the lipid bilayer structure a decidedly thinner peptidoglycan layer. Above this, however, they demonstrate the presence of another membrane (therefore Gram-negative bacteria are double-membrane bacteria) whose structure is however peculiar. In fact, if the layer facing the inside of the cell body is completely identical to the Gram-positive membranes, the outer layer is instead composed of a liposaccharide matrix, the LPS precisely [45]. Therefore, Gram-negative bacteria are bacteria characterized by a double membrane, and for this reason they are also identified as “diderms” and are opposed in this sense to the Gram-positive ones defined therefore as “monoderms”, of which the outermost one is entirely made of LPS. Differently from what it would be easier to think, Gram-positives are not, however, those that are stained with crystal violet (or gentian violet). Similarly, Gram-negatives are not those that do not stain with the same dye. Both, on the other hand, are colored. However, the Gram-positives, when passing into alcohol or acetone, do not lose the crystal violet, while the Gram-negatives, whose LPS is destroyed by this second passage, are discolored [46]. From an evolutionary point of view, it has been recently proposed that bacteria were “born” as diderms. Later, they may have lost the outer layer and survived to the extent that they developed the ability to increase the peptidoglycan layer, a structure which, when thickened, determines protection and replaces the second membrane. In fact, if the second membrane is removed from Gram-negative bacteria, that is, if the LPS is removed, they simply die because the remaining layer is not sufficient to ensure osmotic balance. In fact, the ingress of water from the external environment swells the bacterium until it kills it [47,48].

## 6. The Bacterial Lipopolysaccharide (LPS)

In structural terms, in most of the *Enterobacteriaceae* the LPS is made up of four different parts [49]: lipid A, a real phospholipid, containing on average 6–8 fatty acids and corresponding to the portion of the LPS “stuck” in the external lipid layer of the second membrane of Gram-negative species; a polysaccharide structure called core, which can in turn be divided into an innermost portion (inner core) and into an outermost portion (outer core); last, a long outermost polysaccharide chain, the so-called O antigen, which can contain up to 50 repeated saccharide units and singly is made up of 4–8 monosaccharide residues. Differently, in *taxa* such as *Neisseria*, *Haemophilus*, *Bordetella*, *Acinetobacter* and *Bacteroides*, the LPS “shrinks” to three components, as the O antigen is absent. Finally, in bacteria such as *Chlamydia*, the outer core is also absent and the LPS is therefore reduced to only two components. In terms of structural variability among the different Gram-negative species, this appears to be very high for the O antigen, high for the outer core, moderately high for the inner core and low for lipid A. Lipid A is certainly the most conserved structure within the LPS, however, it can be traced back to three different types, known simply as A (typical of *Escherichia*/*Shigella*, *Campylobacter*, *Haemophilus*), B (typical of *Chromobacterium*) and C (typical of *Rhodospirillum*) [50].

## 7. Toxicity and Immunogenicity of LPS

The toxicity of LPS was first identified by a German scientist (Richard Pfeiffer) at the end of the 19th century. Working with strains of *Vibrio cholerae*, he had previously discovered that their exotoxin, the true etiological factor of cholera, was deactivated with heat. At the same time, however, he had also noticed that, regardless of the exotoxin, if he experimentally inoculated the bacterium “cooked” (dead and without exotoxin) or “torn to pieces” (dead), the animal still showed strong toxicity (fever, hypotension, disseminated intravascular coagulation, septic shock). This toxicity, different from that typical of cholera, was therefore not eliminable with heat and did not require a live pathogen. To distinguish it from exotoxin, Pfeiffer called the substance he believed to be the cause of sepsis “endotoxin.” He later identified the same substance in all *Enterobacteriaceae*. Curiously, despite having very little of “endo” (it is in fact the most peripheral portion of Gram-negative bacteria), LPS is still commonly called endotoxin today [51]. However, LPS is not just a toxin. It is also, for example, the key to entry for those bacteriophages that recognize Gram-negative bacteria as their target [52]. However, as regards the bacterium-host interface, it is above all a powerful immunogen capable of generating immune responses, unfortunately also very “inflammatory”, thanks to the response of populations such as plasma cells, monocytes/macrophages, granulocytes, and APCs (antigen presenting cells). According to some authors, it is even probable that evolution has used the LPS as a personal trainer for immunity, to encode effective responses against Gram-negative bacteria [53]. Although each structural component obviously has its own specific role, the toxicological properties of LPS are mainly due to the structure of lipid A and to that of the inner core. Lipid A would turn out to be the real toxophore. Its presence alone, in fact, determines the release of TNF-α, IL-1 and IL-6 by monocytes. The inner core, on the other hand, would have a certain role in the modulation of the toxic action of lipid A [53]. The immune and inflammatory response evoked by the LPS is due to two different mechanisms. The first, non-specific, is simply due to the lipophilicity of lipid A and therefore its ability to “dissolve” both in cell membranes and in post-prandial fat particles. The second, more specific and more powerful, is mediated by its interaction with the LPS binding protein or LPB [54]. The LPS-LPB complex interacts with CD14. The latter, through the MD-2 (myeloid differentiation factor 2) protein, activates the TLR-4 (Toll-like receptor-4) receptor. This is followed by a cytoplasmic cascade that begins with the release of MyD88 (myeloid differentiation primary response 88) and ends with the up-modulation of NF-kB (nuclear factor kappa-light-chain-enhancer of activated B cells), an intra-nuclear factor capable of generating the transcription and translation of pro-inflammatory cytokines such as TNF-α, IL-1 and IL-6 [55].

## 8. Role of the LPS Derived from Bacteroidetes

At the colonic level, quantitatively speaking, it is not the *Enterobacteriaceae* family that is most responsible for the degree of total Gram-negativity. The group of Bacteroidetes (*Bacteroides* in primis, but also *Parabacteroides*, *Alistipes*, *Rikenella*, *Prevotella*, *Paraprevotella*, *Alloprevotella*, etc.) is in fact on average ten times more consistent [56]. As previously described, the LPS of bacteria belonging to this *phylum*, for example to the genus *Bacteroides*, is structurally different from that of *Escherichia*/*Shigella*, as the O antigen is absent. This difference makes it less efficient in generating the release of inflammatory cytokines and perform less in demonstrating lethality. In in vitro models that measure the release of TNF-α, the LPS from *Bacteroides* spp. performs between 5 and 10 times less than the LPS from Escherichia. In mice, this action gap increases considerably, showing a lethality about 1000 times lower for the LPS from *Bacteroides* than that observed by inoculating the LPS from Escherichia [57]. Despite this difference, in assessing the degree of total toxicity of the colonic-derived LPS, the strong representation of Bacteroidetes cannot be disregarded. Likewise, the presence of the Negativicutes (*Megasphaera*, *Megamonas*, *Acidaminococcus*, *Dialister*, etc.) cannot be ignored. These probable evolutionary bridges between diderms and monoderms are in fact monoderms that possess LPS on the outer membrane. For this reason, they can contribute to the total Gram-negativity of the colonic microbiota [58].

## 9. Gram-Negativity of the Colonic Microbiota and Metabolic Endotoxemia

**Figure 4:**
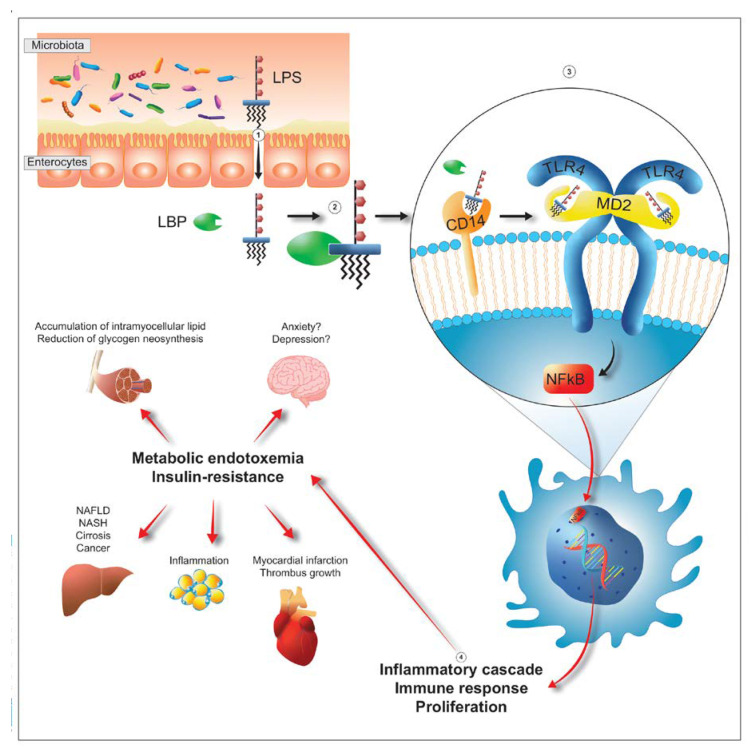
After passing the barrier formed by enterocytes (step 1), LPS interacts with the LPS-binding protein, known as LBP (step 2). The complex then interacts with the CD14 placed on the membrane of the target cells (step 3). Then, CD14 transfers LPS into the receptor pocket of the complex constituted by TLR4 (Toll-like receptor 4) and MD2 (myeloid differentiation-2) triggering a cascade of intracytoplasmic events which culminates with the entry of NFkB (nuclear factor kappa-light-chain-enhancer of activated B cells) into the nucleus and subsequent transcription and translation into proteins (mainly cytokines) with specific inflammatory characteristics (step 4). The presence of these cytokines, feeds phenomena such as metabolic endotoxemia and tissue insulin-resistance (step 5). Both, by acting on the different organs, have different consequences. In the liver, they promote the development of NAFLD (non-alcoholic fatty liver disease), NASH (non-alcoholic steatohepatitis), cirrhosis and cancer. In the striatal-skeletal musculature they promote accumulation of intramyocellular lipid and reduction of glycogen neo-synthesis. In the adipose tissue they promote inflammation. At cardiac and cardio-vascular levels they predispose individuals to an increased risk of myocardial infarction and thrombus growth. Finally, in the brain, they probably promote pathologies such as anxiety and depression.

LPS can be derived from at least five bacterial groups, Proteobacteria, Verrucomicrobioa, Fusobacteria, Bacteroidetes and Negativicutes, and reaches the peripheral circulation through three different routes. It can pass through the enterocyte monolayer by exploiting the possible non-solidity of the tight junctions thus reaching the lamina propria, it can “contact” the lymph node stations simply by exploiting the contractility of the M cells and the presence of abundant lymphocytes underlying them and it can blend with dietary fats and take advantage of the delivery offered by chylomicrons to quickly reach the liver [59,60]. There are numerous situations where the presence in the circulation of LPS of colonic origin (metabolic endotoxemia) probably plays an active role in pathological genesis (Figure 4). For example, in the anxious-depressive syndrome *Enterobacteriaceae* and Bacteroidetes are dominant and in these subjects the metagenomic picture shows a strong propensity of the consortium to express genes aimed at the production of LPS. In addition, plasma analysis shows a greater amount, compared to healthy subjects, of both LPS and zonulin, demonstrating a possible process of absorption of LPS mediated by a more permeable than normal intestine [61]. Colonic-derived LPS can then affect the liver, through the post-prandial fat particles, thanks to a first contact with the CD14 placed on the membrane of Kupffer cells, hepatic macrophages belonging to the reticulo-histiocytic or reticulo-endothelial system, whose inflammatory response reverberates heavily on hepatocytes and stellate cells increasing the risk of evolution to steatosis, fibrosis, cirrhosis and carcinoma [62,63]. Beyond depression and liver disease, metabolic endotoxemia is still effectively involved in an important number of diseases: in diabetes, for example, where the plasma LPS value of colonial origin is from 66 to 235% higher than in controls [64]; in cardiac pathologies, from stable angina to heart attack, where it reaches values respectively 2 and 3 times higher than in healthy controls [65] or in cardio-metabolic diseases, such as obesity and metabolic syndrome, where the risk of cardiovascular accident increases in proportion to the increase in plasma LPS value [66]. The involvement of LPS is also evident in autoimmune diseases, such as rheumatoid arthritis, where an important role is probably played by the LPS of *Prevotella copri* [67], and also in chronic fatigue syndrome [68], in HIV [69], in asthma and atopy, even without a particular correlation with IgE values or with pictures of pronounced eosinophilia [70,71,72]. Finally, a strong correlation was observed in inflammaging, also experimental [73,74], and with the severity of patients in intensive care [75] in which the ratio between Gram-positive and Gram-negative bacteria is certainly higher in controls than in hospitalized patients and is higher, a parameter measured 12 months after initial hospitalization, in the survivors rather than the deceased.

## 10. The Gram-Positive/Gram-Negative Ratio

As highlighted in the study performed on ICU patients [75], the numerical ratio between the relative percentages of the Gram-positive and Gram-negative populations of the colonic microbiota (G+/G) constitutes a possible marker of inflammation. Considering that in standard conditions (adult subject on a Mediterranean diet, in the absence of obvious pathologies and with a Bristol stool score between 3 and 4) the Gram-negative species are on average double the Gram-positive species [20], the ratio assumes, as a reference, values between 0.4 and 0.5. Lower values, tending to 0.1, are therefore due to a greater contribution of Gram-negative species; higher values, tending to 1, are instead due to a greater contribution of Gram-positive species. A very low G+/G− ratio, for example between 0.1 and 0.2, assumes a probably inflammatory condition, in which the LPS potentially released into the system reaches high values, helping to establish, if not already existing, an inflammatory state. Obviously, a high bacterial Gram-negativity does not necessarily imply a state of disease but only a predisposition to the construction of potentially inflammatory pictures mediated by LPS. These pictures of potential inflammation can then be confined to the colonic area or, conversely, permeating the latter and therefore able to reach the periphery (liver, omentum, blood circulation, joints, etc.). The analysis of parameters such as fecal calprotectin and plasma zonulin could help distinguish between these two possibilities. Calprotectin, a cytosol protein released predominantly by neutrophils, is commonly tested for in inflammatory bowel disease. Although detectable in various body fluids at concentrations proportional to the degree of inflammation, in the stool it reaches levels about six times higher than those detectable in the blood. The presence of fecal calprotectin therefore reflects intestinal inflammation and excludes the presence of functional disorders [76]. Zonulin, a protein involved in the modulation of intestinal permeability, acts by opening the tight junctions between the cells of the digestive tract wall. Identified by the Italian researcher Alessio Fasano and his team operating at the Maryland School of Medicine, it is now considered the best indicator of possible leaky-gut syndrome [77]. Its detection, coupled with a very low G+/G− ratio, could therefore suggest the possible existence of a systemic inflammatory state.

## 11. The Prevotella/Bacteroides Ratio

Another potential biomarker, easy to derive and easy to interpret, is the *Prevotella*/*Bacteroides* (P/B) ratio. Obtained simply by reporting the relative percentage of *Prevotella* (+U. m. of *Prevotellaceae*) to the numerator and the percentage of *Bacteroides* to the denominator, it is possible to obtain a parameter capable of identifying overweight or obese subjects who should respond better to a low-calorie diet when this is also particularly rich in vegetable fiber. In industrialized societies the frequency of subjects in relation to their P/B ratio demonstrates a bimodal graph in which the first curve, with a higher frequency, falls for the subjects with a low P/B ratio, and the second, with lower frequency, shows subjects with a high P/B ratio [78]. As published, subjects with a high P/B ratio would have the best chance of losing weight if the diet, certainly low-calorie, was particularly rich in vegetable fiber [79,80]. Beyond the purely caloric aspects, the main feature of these diets is the rich contribution of polysaccharide fiber, which cannot be fermented at the level of the small intestine and is well metabolized in the colonic area. This “distal” digestion is certainly due to the saccharolytic capacities of the bacteria, which in the colon transform the fiber, thanks to their role of final electron acceptors, into short chain fatty acids (SCFA). Fibers like fructo-oligosaccharides, galacto-oligosaccharides, xylo-saccharides, etc. are fermented, according to the type of bacterium involved, mainly to acetate, propionate and butyrate and, in smaller quantities, to succinate and lactate. Therefore, even if not metabolized in the small intestine and therefore not absorbed by it as monosaccharides, these fibers can still make their own contribution to the caloric balance of the meal. Specifically, if what is assimilated in the small intestine as hexose generates a caloric balance of 3.9 kcal/g, a value of 1.3 kcal/g should be calculated for these fibers [81]. The overall amount of SCFA that develops in subjects with an elevated *Prevotella* colonic profile is certainly greater than that which develops in subjects with low *Prevotella* presence [82]. Therefore, even in consideration of the low average caloric profile obtainable, the *Prevotella* dominance increases its production. However, if one evaluates individually the contribution that the two different dominances can determine in terms of SCFA, it can be observed that propionate is the one able to make the difference giving the greatest contribution, almost total, to the calculation of the total SCFA. Regardless of dominance, in subjects on a low-calorie diet we have a balance of potentially absorbable calories in the small intestine lower than the value of the pre-diet period. This phenomenon is largely responsible for the weight loss observed in adherent subjects. However, it is precisely the adherence that can make the difference between the *Prevotella* and the *Bacteroides* dominances. This is certainly linked to psychological aspects but also to phenomena, known to anyone who has ever undergone a diet, such as the perception of hunger. A low-calorie diet also capable of feeding the phenomenon of satiety would therefore have a better chance of success precisely by virtue of the subject’s lower perception of hunger. In fact, at least experimentally, in very controlled microbiota models, propionate is produced and released in the case of strong preponderance of *Prevotella* at values even three times higher than those detected in the *Bacteroides* dominance [83]. As it is well-known, propionate, after having bypassed the enterocyte line, enters the hepatic gluconeogenesis circuit, and probably contributes to determining a satiating effect [36,37]. *Bacteroides*, both as saccharolytic and as Bacteroidetes, is a taxon obviously described to produce primarily propionate, but the biodiversity and functional redundancy observed in the *Bacteroides* dominance are certainly lower than those evidenced in the *Prevotella* dominance [38]. Therefore, with the same bacterial quantity and the same amount of ingested fiber, the *Prevotella* dominance is likely more efficient in the production of propionate. Furthermore, it must be considered that in the *Bacteroides* dominance it is more common to detect an important presence of Firmicutes, and in fact, in the case, for example, of xylo-saccharides from cereals and sorghum, the butyrate produced is in fact greater in the *Bacteroides* dominance [83], and as shown when describing the F/B ratio, the ability to extract calories by transforming them into butyrate is, on the contrary, a parameter that correlates with overweight and obesity.

## 12. The *Fusobacterium nucleatum*/*Faecalibacterium prausnitzii* Ratio

Some authors have recently described the possibility that some gut species, such as *Fusobacterium nucleatum* (*Fn*), may behave within the microbiota as “oncobacteria”, which can promote colon neoplasms [84]. There is also the opposite possibility: some bacteria may apparently exercise an antitumor role, as in the case of *Holdemanella biformis*, considered a protective species (also) due to its capability to produce butyrate [85]. Since the genus *Holdemanella* in colonic consortia is found with a rather low frequency, some authors have proposed to detect another, much more frequently detectable, butyrate-producing taxon: *Faecalibacterium prausnitzii* (*Fp*) [86]. In particular, the analysis of the *Fn*/*Fp* ratio has proved useful in intercepting subjects with colon adenomas, chronic inflammatory bowel diseases (IBD) and colorectal carcinomas (CRC), differentiating them from healthy subjects and from subjects with non-gut neoplastic lesions. The study [87], performed on almost 1000 subjects, has shown that the *Fn*/*Fp* ratio is low, less than 1, in healthy controls and in subjects with non-intestinal diseases, while it is higher in subjects diagnosed with IBD, adenomas and CCR. In the latter case, moreover, the increase in the *Fn*/*Fp* ratio would be proportional to the increase in the severity of the neoplasm. The ability to intercept CRC situations, thanks to the *Fn*/*Fp* ratio, would demonstrate a specificity of 94.5% and a sensitivity of 71.28%. A different ratio, between *Fn* and the taxon *Bifidobacterium* (*Fn*/*Bb*), would instead be able to intercept only subjects at risk of CRC without however highlighting subjects with the possible presence of adenomas or diagnosis of IBD. This second ratio would therefore be less useful in intercepting severely dysbiotic situations in a broader sense. However, if the ability to intercept the initial stages of CCR is evaluated, the best performance is that obtained using the *Fn*/*Bb* ratio (specificity: 81.9%, sensitivity: 75%). In consideration of this second aspect, it could be strategic to observe both indices. One of the possible explanations for the poor detection of *Fp* and *Bb* in the presence of high percentages of *Fn* is provided by the lack of in vitro growth of both *Fp* and *Bb* in the presence of the culture supernatant obtained from *Fn* [87]. The latter is a well-known producer of bacteriocins [88] to which both genera, *Faecalibacterium* and *Bifidobacterium*, could be sensitive. Regardless of the importance of the *Fn/Fp* and *Fn/Bb* indices, the simple detection of *Fn* by analyzing the colonic microbiota could be, probably even alone, a good marker of the possible presence of colon cancer. A recent meta-analysis performed on 4500 subjects, including controls and patients with colon adenoma or carcinoma, showed a specificity of 76% and a sensitivity of 71% in relation to the possibility of intercepting CCR cases and, in relation to the diagnosis of adenoma, a specificity of 73% and a sensitivity of 36% [89]. These results resemble the ones got by Guo S. et al. [87]. In their study the authors observed in fact lower values of specificity and sensitivity by using the “simple” detection of *Fn* rather than by using the ratio *Fn/Fp*. The results, even if important, do not minimize the negative prediction that *Fusobacterium* detection could, or should, rise.

## 13. Conclusions

Recent advances in technical capabilities for analyzing human gut microbiota have led to an explosion of microbiome research. This has surely promoted a strong interest among clinicians in applying these techniques to their patients to improve the capability of intercepting possible diseases. In this review, written in a narrative way and with the approach of simplifying some excessive complexities typical of the gut microbiota, some possible markers have been taken into consideration in trying to evaluate if and how they could be contributing to a better understanding of patient conditions. A particular focus has been given to some ratios, easily deducible after analyzing the stool microbiota, such as the F/B, the P/B, the G+/G− and the *Fn*/*Fp* ones. Certainly, these markers need further investigation and still have some limitations. However, they show possible promise for the future, and to keep debating them, as it has been done in this narrative review, could prompt researchers to make new and more accurate attempts to improve the techniques necessary to ameliorate them and could accelerate the path toward their routine clinical application.

However, as of now these parameters could become part of the routine approaches in outpatient and inpatient settings. Obviously, first it would be necessary to optimize as much as possible a series of analytical parameters that are still too little considered as important variables capable of modifying, at least in part, the results. This optimization would make it possible to obtain a greater degree of uniformity and overlap of the results obtained, even if from different laboratories. For example, too many laboratories rely on too low sequence homologies [90,91]. It would then be advisable for everyone to adopt 100% sequence homologies, to change from the concept of OTU (operational taxonomic unit) to the more precise one of ASV (amplicon sequence variant) [92,93]. Obviously, this should be done together with a better profiling of the filtering systems to avoid as much as possible having to interpret sequences resulting from errors as real reads [94]. To achieve this, it would be necessary to try to standardize the filtering methods or, at least, to validate the possible bioequivalence of different systems. Another problem concerns the updating of the databases used in the taxonomic attribution of the obtained reads. Too often, laboratories refer to databases that are not sufficiently up to date [95,96]. Obviously, many others would be the variables to be standardized (DNA extraction methods or the regions of the 16S rRNA gene to be analyzed), but at least looking for uniformity in the sequence homology, in the cut-off filtering of the reads and in the databases used could be considered a good step forward in this regard. Once a greater degree of analytical uniformity has been achieved, the parameters discussed in this review could then actually be used in clinical practice. Surely, the F/B ratio could be used to decide more precisely the value of calories to be set in a weight loss diet, taking into greater consideration the calories derived from the SCFA [97]. The same parameter could also be used, during the same therapy, to evaluate the trend of the caloric extraction capacity during the continuation of the diet. The G+/G− ratio, with due assessments to be performed on taxa that possess a membrane with LPS, should be used to assess the inflammatory potential (pro-TNF-α, pro-IL-1β, pro-IL-6, etc.) of the microbiota. To better understand to what extent the inflammatory power of the microbiota could then be transferred outside the intestinal area (liver, muscles, adipose tissue, brain, etc.), the analysis of fecal zonulin could be of help [61,98]. Regardless of zonulin, a strong Gram-negativity should always be considered potentially inflammatory and therefore managed with a nutritional intervention (for example with the reduction of dietary fat) and/or with a probiotic and prebiotic. The test should be carefully evaluated in obese, diabetic and insulin-resistant patients and those on a ketogenic diet. In this case, the monitoring of Gram-negativity could suggest a change to a less inflammatory diet such as the Mediterranean one [99]. Based on a recent publication that demonstrates how intestinal microbiota-derived LPS can contribute to anxious-depressive manifestation [100], analysis of the G+/G− ratio could help the physician in deciding whether to set up a conventional-only therapy (with anti-depressants and/or anxiolytics) or whether to intervene also on the diet trying to reduce the total Gram-negativity. Even if the *Prevotella* content in people of industrialized countries is certainly modest, the P/B ratio could also be advantageously applicable in diets. For example, a high P/B ratio could suggest the possible success of a diet rich in fiber and could reduce the use of the ketogenic diet, likely inflammatory, which perhaps too often is applied by physicians and nutritionists to achieve safer weight loss. Finally, the relationship between *Fusobacterium* and *Faecalibacterium* or between *Fusobacterium* and *Bifidobacterium* could help, as is done with the analysis of stool occult blood or of calprotectin [101], to intercept situations that could evolve to adenomas or carcinomas of the colon or to inflammatory bowel disease. Surely, even the simplest detection of *Fusobacterium* alone should sound like an “alarm bell”. However, as illustrated in the relative paragraph, the presence of an “aggressor” (*Fusobacterium*) is probably less dangerous (therefore less prodromal to the disease) in the presence of a “protector” (*Faecalibacterium* or *Bifidobacterium*). As the specificity and sensitivity results show, analysis of the relationship between *Fusobacterium* and *Faecalibacterium* (or *Bifidobacterium*), rather than just the detection of *Fusobacterium*, demonstrates more significant results.

In conclusion, altered gut bacterial compositions have been associated with the predisposition and/or the pathogenesis of many diseases. Some parameters (F/B, G+/G−, P/B and *Fn*/*Fp*) could be used to better classify and understand these altered consortia and to elaborate a possible plan of intervention.
